# Pectoralis Muscle Mass on Chest CT at Admission Predicts Prognosis in Patients with Pneumonia

**DOI:** 10.1155/2021/3396950

**Published:** 2021-12-06

**Authors:** Ryohsuke Yokosuka, Ryosuke Imai, Shosei Ro, Manabu Murakami, Kohei Okafuji, Atsushi Kitamura, Yutaka Tomishima, Torahiko Jinta, Naoki Nishimura, Tomohide Tamura, Osamu Takahashi

**Affiliations:** ^1^Department of Internal Medicine, St. Luke's International Hospital, Tokyo, Japan; ^2^Department of Pulmonary Medicine, Thoracic Center, St. Luke's International Hospital, Tokyo, Japan; ^3^Graduate School of Public Health, St. Luke's International University, Tokyo, Japan

## Abstract

**Methods:**

A retrospective cohort study was performed in patients aged 18 years or older with pneumonia who underwent chest CT within 24 hours of admission between April 2014 and March 2019. We measured the thickness, area, and volume of the pectoralis major and minor muscles at the level of the aortic arch. Factors associated with mortality were examined using logistic regression analysis.

**Results:**

A total of 483 patients (mean age 77 ± 14 years, 300 men (62%)) were included, and fifty-one patients (11%) died during admission. In univariate analysis, decreased thickness, area, and volume of the pectoralis major and minor muscles were associated with higher in-hospital mortality. Multivariate analysis with adjustment for age, gender, serum albumin, and A-DROP revealed that thinner pectoralis major and minor muscles were independent factors of poor prognosis (odds ratio: 0.878, 95% confidence interval (CI): 0.783–0.985, *P*=0.026 and odds ratio: 0.842, 95% CI: 0.733–0.968, *P*=0.016, respectively). Approximately 25% of the patients died when the pectoralis minor muscle thickness was 5 mm or less, and no patients died when it was 15 mm or more.

**Conclusion:**

The pectoralis muscle mass may be an independent prognostic factor in hospitalized patients with pneumonia.

## 1. Introduction

Pneumonia is one of the most common causes of death in Japan, and prognosis at the time of hospitalization is important. A-DROP and the Pneumonia Severity Index (PSI), which is calculated by combining multiple factors such as patient backgrounds, are useful for predicting the prognosis of pneumonia, but a simpler and intuitive index for predicting prognosis has not been established [1].

On the other hand, sarcopenia is a syndrome characterized by a decrease in the quantity and quality of skeletal muscle throughout the body, which has recently been proposed to be associated with prognosis in a variety of diseases [2]. Recent studies suggest that not only the quantity but also the quality of muscle is one of the important factors in sarcopenia [3]. As a method of assessing sarcopenia, computed tomography (CT) is used to measure the volume of the pectoral muscles and erector spinae muscles [4–6], and in fact, sarcopenia has been shown to be associated with prognosis in ICU patients and gastric cancer surgery [7, 8]. Some studies have examined the relationship between muscle mass measured by CT and prognosis in idiopathic pulmonary fibrosis, left ventricular assist device implantation, and COVID-19 [3, 9–12]. However, the association of muscle mass on CT with prognosis in patients with pneumonia is not clear. Inpatients with pneumonia often undergo chest CT, which allows measurement of pectoralis muscle mass.

The purpose of this study was to investigate the relationship between pectoralis muscle mass on chest CT and in-hospital mortality in patients hospitalized with pneumonia and to find a useful index for predicting the prognosis of pneumonia.

## 2. Methods

A retrospective cohort study was conducted on patients aged 18 years and older who were admitted to the Department of Pulmonary Medicine at our hospital with a diagnosis of pneumonia between April 1, 2014, and March 31, 2019, and who had a chest CT scan within 24 hours of admission. Patients who had not had a chest CT performed within 24 hours of their admission, patients with acute exacerbation of interstitial lung disease and chronic obstructive pulmonary disease (COPD), and patients whose images were not clear enough to accurately measure pectoralis muscle mass were excluded.

**Figure 1:**
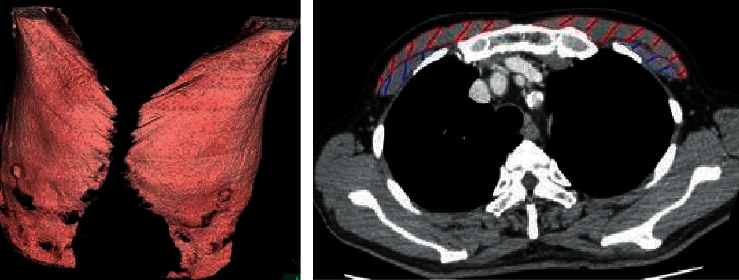
Pectoralis muscles and 3D reconstruction. Red: pectoralis major muscle; blue: pectoralis minor muscle.

Patient background (gender, age, height, weight, smoking history, and medical history), laboratory tests, arterial blood gas tests, A-DROP, PSI, oxygen devices at the time of admission, and clinical outcome were extracted from the medical records of the patients. In addition, the thickness, area, and volume of the pectoralis major and minor muscles, respectively, were measured by chest CT scan on admission. The image analysis software (Fujifilm SYNAPSE VINCENT) was used to measure the maximum thickness and area of the pectoralis major and pectoralis minor muscles on CT slices of the aortic arch at its apex, as well as the total volume of the pectoralis major and pectoralis minor muscles combined, and the average values of the left and right sides were used for analysis (Figure 1). It has been shown that this level gives the best results and is highly reproducible [2, 13]. The measurement method was based on the previous study [7].

Patients were divided into two groups: survivor and nonsurvivor groups, during hospitalization. Categorical variables were compared using the chi-square test. Continuous variables were compared using Student's *t*-test. To identify independent risk factors for in-hospital mortality, the thickness, area, and volume of the pectoralis major and minor muscles and already known prognostic factors such as age, sex, albumin, and A-DROP were included in the multivariable logistic regression model. All statistical tests were two-tailed, and significance was accepted at *P* ≤ 0.05. All statistical analyses were performed with EZR (Saitama Medical Center, Jichi Medical University, Saitama, Japan), which is a graphical user interface for *R* (The *R* Foundation for Statistical Computing, Vienna, Austria, version 4.0.4). More precisely, it is a modified version of *R* Commander (version 1.54) designed to add statistical functions frequently used in biostatistics [14]. This study was conducted with the approval of the Ethics Committee of St. Luke's International Hospital (Reference number: 19-J011).

## 3. Results

**Figure 2:**
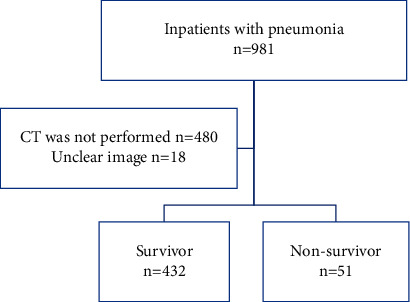
Target patients.

A total of 981 patients with pneumonia were admitted during the period, of which 480 patients who had not had a CT scan within 24 hours of admission and 18 patients whose muscle mass could not be measured accurately because of the lack of clarity of the images were excluded. Four hundred and eighty-three patients were included in the study. Fifty-one patients (11%) died during admission (Figure 2).

The clinical characteristics of the eligible patients are shown in Table 1. The mean age ± standard deviation was 77 ± 14 years, with 300 (62%) males. In older patients, interstitial lung disease comorbidity tended to be higher in the nonsurvivor group, and body mass index (BMI) tended to be lower. There were no differences in gender, other comorbidities, or smoking history between the two groups. The use of oxygen devices such as noninvasive ventilation (NIV) and high-flow nasal cannula oxygen therapy (HFNC) was more common in the nonsurvivor group, and serum albumin levels tended to be lower. There was no significant difference in the inflammatory response; A-DROP and PSI tended to be higher in the nonsurvivor death group (Table 1).

The volume of the total pectoralis muscles tended to be smaller in the nonsurvivor group compared to the survivor group. A similar trend was seen in the area and the thickness of the pectoralis major and minor muscles (Table 2).

**Figure 3:**
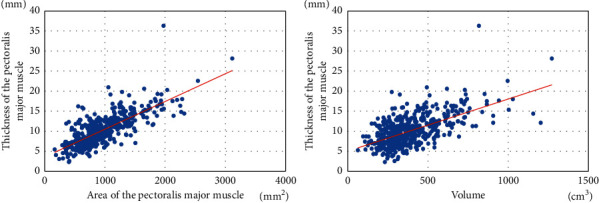
Correlation of area and volume with the thickness of the pectoralis major muscle.

**Figure 4:**
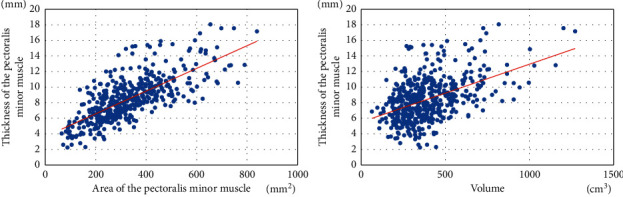
Correlation of area and volume with the thickness of the pectoralis minor muscle.

The correlation between thickness and area/volume was analyzed (Figures 3 and 4). The correlation coefficient between the thickness of the pectoralis major muscle and the area/volume of the pectoralis major muscle was 0.756 and 0.609, respectively, which showed a constant correlation. The thickness of the pectoralis minor muscle also showed a constant correlation with area and volume (pectoralis minor muscle thickness and area and volume, *r* = 0.725 and 0.478), suggesting that the thickness of the pectoralis major and pectoralis minor muscles may be a substitute for area and volume.

Multivariate analysis of age, gender, and prognostic variables (serum albumin level and A-DROP) showed that albumin level and pectoralis major and minor muscle thickness were independently associated with in-hospital mortality. In particular, there was a stronger association with the thickness of the pectoralis minor muscle (Table 3).

**Figure 5:**
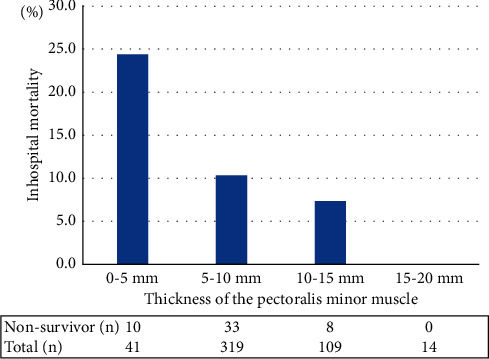
Thickness of the pectoralis minor muscle and in-hospital mortality.

The relationship between pectoralis minor muscle thickness and in-hospital mortality is shown in Figure 5, where the in-hospital mortality rate was calculated for each of the four categories at 5 mm. Nearly 25% of patients died in-hospital at 5 mm or less, and there were no in-hospital deaths at 15 mm or more. The thicker the pectoralis minor muscle, the less in-hospital mortality was observed.

## 4. Discussion

In this study, patients with pneumonia whose pectoralis minor muscle was thin on chest CT scan at admission tended to have higher mortality during hospitalization. The prognosis of pneumonia can be predicted by measuring the thickness of the pectoralis minor muscle, and chest CT scans are often performed in the management of pneumonia. It provides a visual prognosis and is useful for explaining to patients and their families, without the need to combine multiple factors, as is the case with A-DROP and PSI. The visual recognition of thin pectoral muscles allows patients and their families to more intuitively recognize the high mortality rate from pneumonia.

Several previous studies have reported an association between sarcopenia and prognosis [15–26]. Jaitovich et al. reported that, in patients admitted to the ICU, the area of the thoracic muscle on CT at admission was associated with an acute stage survival rate [7]. Poor skeletal muscle quality at admission to the ICU was associated with death at 6 months in patients who were managed on a ventilator [27]. Another study reported the lower the CT value, the worse the muscle quality [28]. There have been reports on the risk of perioperative complications, and sarcopenia based on muscle mass in the third lumbar vertebra and other factors was associated with 30-day mortality and the occurrence of major postoperative complications in patients undergoing abdominal surgery [29]. In COPD, the area of the pectoralis major muscle has been shown to be associated with prognosis and disease severity, and the possibility of progression of frailty due to the disease itself has also been pointed out [2]. The present study is the first to show a possible association between chest muscle mass measured by CT and in-hospital mortality in hospitalized patients with pneumonia.

The pectoralis major and pectoralis minor muscles are both known to be associated with respiration, and both are thought to assist in inspiration by pulling the rib cage upward and outward [30]. The amount of the pectoralis major and minor muscles may reflect the breathing reserve [31], which may be associated with in-hospital mortality in patients with pneumonia. Patients with sarcopenia may be more prone to respiratory muscle fatigue during severe pneumonia.

In this study, there was a greater association with in-hospital mortality in the pectoralis minor muscle than in the pectoralis major muscle. This difference was thought to be related to the fact that the pectoralis major muscle is predominantly fast muscle (type II), which is responsible for upper arm exercises, and the pectoralis minor muscle is predominantly slow muscle (type I), which is responsible for respiratory support [30]. In other words, it is possible that the pectoralis minor muscle is more reflective of respiratory status and has the ability to expectorate. In fact, one study has shown that the pectoralis minor muscle is more related to lung expansion than the pectoralis major muscle [31].

The limitations of the study include the lack of external validity because it was a single-center, retrospective cohort study. A larger, multicenter, prospective study is needed to generalize the results of this study. In addition, the study population was limited to inpatients who were admitted to the hospital after CT scan, which may have limited the population to critically ill patients. There is a possibility that the patient has a comorbidity other than pneumonia, or that the patient has pneumonia that is difficult to diagnose on a simple chest picture.

As a future issue, this study used muscle mass on CT as an indicator of sarcopenia, but it may be necessary to evaluate the relationship with other commonly used indicators of frailty, such as the Clinical Frailty Scale [32].

The amount of pectoral muscle varies with age, gender, and body size. Therefore, comparing them by absolute value may lead to bias. It may be necessary to compensate by ideal weight or to take body shape into account.

However, if this study can be used as a pilot study to examine the relationship between pectoral muscles and prognosis or sarcopenia/frailty and prognosis using big data or a prospective trial, the prognosis of pneumonia may be changed by intervening in patients earlier.

## 5. Conclusion

Thoracic muscle mass has been shown to be a possible independent prognostic factor in hospitalized pneumonia patients.

## Figures and Tables

**Table 1:** Patient characteristics.

		Total	Survivors	Nonsurvivors	*P* value
Age (yr)		77 ± 14	76 ± 14	84 ± 8	<0.001
Men		300 (62)	269 (6)	31 (61)	0.957
BMI (cm/kg^2^)		20.7 ± 4.3	20.9 ± 4.3	18.5 ± 3.4	<0.001

Comorbidity	Malignancy	135 (27.9)	115 (26.6)	20 (39.2)	0.084
	COPD	68 (14.0)	58 (13.4)	10 (19.6)	0.323
	Asthma	33 (6.8)	32 (7.4)	1 (2.0)	0.220
	Interstitial lung disease	44 (9.1)	34 (7.9)	10 (19.6)	0.013

Smoking history	Nonsmoker	144 (29.8)	131 (50.6)	13 (39.4)	0.342
	Current smoker	48 (9.9)	43 (16.6)	5 (15.2)	
	Past smoker	100 (20.7)	85 (32.8)	15 (45.5)	

Device	Intubation	11 (2.3)	9 (2.1)	2 (3.9)	0.737
	NIV	36 (7.5)	22 (5.1)	14 (27.5)	<0.001
	HFNC	67 (13.9)	40 (9.3)	27 (52.9)	<0.001

Laboratory data	WBC (×10^3^/*μ*l)	11.3 ± 5.4	11.3 ± 5.3	11.8 ± 6.3	0.537
	Albumin (mg/dl)	3.3 ± 0.6	3.3 ± 0.6	2.9 ± 0.4	<0.001
	CRP (mg/dl)	11.4 ± 9.7	11.1 ± 9.7	13.4 ± 9.2	0.122

A-DROP	0	32 (6.6)	32 (7.4)	0 (0)	<0.001
	1	69 (14.3)	69 (16.0)	0 (0)	
	2	123 (25.5)	113 (26.2)	10 (19.6)	
	3	150 (31.1)	141 (32.6)	9 (17.6)	
	4	92 (19.0)	68 (15.7)	24 (47.1)	
	5	17 (3.5)	9 (2.1)	8 (15.7)	

PSI	1	4 (1.4)	4 (1.6)	0 (0)	<0.001
	2	8 (2.8)	8 (3.3)	0 (0)	
	3	28 (9.9)	28 (11.5)	0 (0)	
	4	98 (34.8)	92 (37.9)	6 (15.4)	
	5	144 (51.1)	111 (45.7)	33 (84.6)	

Continuous variables are expressed as mean ± SD and compared by using Student's *t*-test. Categorical data are expressed as number (%) and compared by using the chi-square test. BMI = body mass index; COPD = chronic obstructive pulmonary disease; PSI = Pneumonia Severity Index; NIV = noninvasive ventilation; HFNC = high-flow nasal cannula; WBC = white Blood cell; CRP = C-reactive protein.

**Table 2:** Pectoralis muscle thickness, area, and volume of survivors and nonsurvivors.

	Total	Survivors	Nonsurvivors	*P* value
Volume (cm^3^)	367.9 ± 165.5	375.8 ± 170.0	301.4 ± 101.8	0.002
*Area* (mm^2^)				
Pectoralis major muscles	950.5 ± 413.9	975.0 ± 422.1	743.3 ± 267.4	<0.001
Pectoralis minor muscles	328.0 ± 136.9	337.5 ± 138.8	247.8 ± 86.8	<0.001

*Thickness* (mm)				
Pectoralis major muscles	10.2 ± 3.8	10.4 ± 3.9	8.4 ± 2.6	<0.001
Pectoralis minor muscles	8.4 ± 2.8	8.6 ± 2.8	7.1 ± 2.3	<0.001

Continuous variables are expressed as mean ± SD and compared by using Student's *t*-test.

**Table 3:** Multivariate logistic regression analysis for prognostic factors associated with in-hospital mortality.

	Odds ratio	95% CI	*P* value
Age	1.030	0.992–1.070	0.119
Sex	1.180	0.599–2.310	0.638
Albumin	0.277	0.152–0.503	<0.001
A-DROP 3 points or more	1.920	0.836–4.410	0.124
Thickness of pectoralis major muscles	0.878	0.783–0.985	0.026

	Odds ratio	95% CI	*P* value
Age	1.030	0.992–1.070	0.116
Sex	1.090	0.567–2.110	0.788
Albumin	0.277	0.153–0.499	<0.001
A-DROP 3 points or more	2.010	0.875–4.630	0.100
Thickness of pectoralis minor muscles	0.842	0.733–0.968	0.016

95% CI = 95% confidence interval.

## Data Availability

The data used to support the findings of this study are available from the corresponding author upon request.

## Abbreviations

BMI: Body mass index
CI: Confidence interval
COPD: Chronic obstructive pulmonary disease
CT: Computed tomography
HFNC: High-flow nasal cannula oxygen therapy
ICU: Intensive care unit
NIV: Noninvasive ventilation
PSI: Pneumonia Severity Index
SD: Standard deviation.
